# Tasting with Feelings: Socioeconomic Differences in Children’s Emotional and Sensory Description of Vegetables

**DOI:** 10.3390/foods15010126

**Published:** 2026-01-01

**Authors:** Karinna Estay, Victor Escalona, Francisca Escobar

**Affiliations:** 1 Departamento de Agroindustria y Enología, Facultad de Ciencias Agronómicas, Universidad de Chile, Santa Rosa 11315, La Pintana 8820808, Chile; francisca.escobar.c@ug.uchile.cl; 2 Departamento de Producción Agrícola, Facultad de Ciencias Agronómicas, Universidad de Chile, Santa Rosa 11315, La Pintana 8820808, Chile; vescalona@uchile.cl

**Keywords:** Check-All-That-Apply, vegetable acceptance, socioeconomic status, sensory evaluation, emojis

## Abstract

Vegetable consumption in childhood remains below recommendations worldwide, particularly in disadvantaged socioeconomic groups. Building on prior work showing no socioeconomic status (SES) differences in children’s liking of familiar vegetables, this study examined whether their sensory and emotional descriptions vary by SES and how these relate to liking beyond hedonic ratings. A total of 363 Chilean fourth graders (9–10 years) from five SES groups evaluated eight vegetables at school. For each sample, children rated overall liking (7-point facial hedonic scale) and completed two CATA (Check-All-That-Apply) tasks: a child-derived sensory list (13 terms) and a validated emoji-based emotion list (33 items). Data were analyzed using Cochran’s Q tests, correspondence analyses, and mean-impact analyses. The use and diversity of sensory and emotional descriptors differed significantly between socioeconomic groups (*p* < 0.05): children from higher SES levels employed a broader and more differentiated vocabulary, while those from lower SES backgrounds used fewer significant terms. Across the sample, juicy, fresh, and mild flavors increased liking, whereas strong aroma decreased it (*p* < 0.05); positive emojis increased liking, whereas negative and neutral ones had no effect. These findings reveal that perceptual and affective representations are socially patterned, underscoring the need to foster sensory–affective literacy in lower-SES contexts.

## 1. Introduction

Vegetable intake during childhood is essential for preventing chronic diseases and promoting long-term health, including cardiovascular disease, certain cancers, type 2 diabetes, and obesity [1,2,3,4]. Despite these benefits, most children worldwide do not consume the minimum recommended amount of vegetables, as consistently reported across regions [5,6,7,8,9]. In 23 countries participating in the WHO European Childhood Obesity Surveillance Initiative, fewer than 25% of 6–9-year-olds consume vegetables daily [10]. In the United States and in many European countries, fewer than 10% of children eat five portions a day [11,12]. In Chile, according to the last National Health Survey, children aged 6–13 consume on average just 167 g of vegetables per day, with children from more vulnerable groups showing even lower consumption levels [13].

Socioeconomic status (SES) is a well-established determinant of dietary quality and health. Evidence from many parts of the world shows marked differences in childhood obesity rates between socioeconomic groups, a gap that, in the case of the United States and Chile, reaches as much as 20 percentage points [14,15]. Lower-SES children tend to have poorer diet quality, with lower intake of nutrient-dense foods such as vegetables.

Vegetables are often mentioned among the least preferred foods by children [16]. This can be explained by their sensory profile, characterized by low energy density and bitterness, which contrasts with children’s innate preference for sweet and calorie-rich foods [17,18]. The development of familiarity can help with vegetable acceptance in childhood; repeated exposure and positive experiences have been shown to improve acceptance [19,20]. However, opportunities to develop familiarity are also unequally distributed; children from lower-SES backgrounds may be exposed to vegetables less frequently and to a narrower variety [21,22].

Beyond taste, emotions can play a pivotal role in shaping food choices. Existing literature indicates that emotions elicited during food consumption can predict choice more accurately than liking alone, and combining emotional with hedonic measures provides the greatest predictive power [23]. While research increasingly recognizes the importance of these affective responses [24], their role in children’s vegetable acceptance—particularly across SES—remains largely underexplored.

A recent study with Chilean schoolchildren found no significant SES-based differences in hedonic ratings for the sensory evaluation of a set of familiar vegetables [25], suggesting that reduced intake among lower-SES children may not stem from taste rejection alone. Building upon that work, the present study explores a complementary dimension of the same phenomenon by examining how children perceive and emotionally describe familiar vegetables. This focus on the expressive and affective aspects of vegetable perception aims to deepen our understanding of the perceptual mechanisms that may contribute to socioeconomic differences in vegetable acceptance.

The Check-All-That-Apply (CATA) method offers a practical approach to capturing consumers’ sensory and emotional descriptions, allowing participants to select multiple attributes that characterize their experience of a given food [26]. It has been validated for use with children across different age groups and cultural contexts [25,27] and is applied with both verbal descriptors [28] and emoji-based emotional cues [29]. Emoji-based CATA has proven reliable for capturing children’s emotional reactions to foods across cultures [30], and affective profiles derived from it can distinguish between products even when liking scores are similar [29,31]. This supports the potential use of this tool to evaluate the sensory perception of vegetables in children across different socioeconomic backgrounds. Moreover, complementing the sensory perception with the emotional profiling of vegetables by children could give a better understanding of vegetable acceptance in childhood.

Despite the growing interest in understanding children’s sensory experiences and preferences toward vegetables, research addressing the social inequalities that shape these perceptions remains scarce. To our knowledge, no studies have examined emotional and sensory descriptions of vegetables by children across SES. This study addresses this gap, analyzing also the number and type of descriptors used by children from different socioeconomic backgrounds when evaluating familiar vegetables. We hypothesize that children from higher-SES contexts will employ a broader and more positively toned vocabulary, while those from lower-SES settings will use fewer descriptors and more negative connotations. By linking these expressive patterns to hedonic ratings, this work aims to identify perceptual and affective barriers that may contribute to low vegetable intake in vulnerable groups.

## 2. Materials and Methods

The present work expands upon data previously reported by Estay and Escalona (2025) [22], which analyzed children’s hedonic responses to familiar vegetables and compared the acceptability of raw and cooked versions of the same samples. In the current study, we examined the complementary sensory and emotional dimensions of those evaluations using the Check-All-That-Apply (CATA) approach with both words and emojis.

### 2.1. Participants and Sampling Design

A total of 363 children aged 9 to 10 years participated in this study. This age group was selected to ensure sufficient cognitive and literacy skills to understand the evaluation tasks and complete the questionnaire independently using tablets. Participants were enrolled in fourth grade across a diverse range of public and private schools located in urban districts of Santiago, Chile.

The sampling strategy was designed to ensure balanced representation across the five socioeconomic status (SES) groups defined by the Chilean Education Quality Measurement System (SIMCE): low, low–medium, medium, medium–high, and high. This national classification assigns all schools in the country to one of these five SES categories—all of which were included in the present study—based on a combination of indicators, including parental education levels, household income, and the School Vulnerability Index (IVE). The IVE is a widely used policy metric that reflects structural and contextual factors such as poverty, unemployment, infrastructure quality, and access to basic services, on a scale from 0 (no vulnerability) to 100 (maximum vulnerability) [32].

A total of 16 schools were involved in the study. Fifteen schools, three from each SES category, were selected for the main data collection. An additional school from the low SES group was used to conduct a pilot test, allowing the refinement of evaluation procedures and logistical protocols. In each participating school, one entire fourth-grade class was invited to participate. Descriptions of the participants by GSE, school, and sex are shown in Table 1.

Children were eligible if they were in fourth grade, had no reported food allergies (as declared by parents), and provided both parental informed consent and their own written assent. All data was collected during school hours, in coordination with each school. The study was approved by the Ethics Committee of the University of Chile (Approval Act 011/2022).

### 2.2. Vegetable Samples

Children evaluated eight familiar vegetables, defined as those commonly consumed and culturally recognized by Chilean school-aged children: tomato, lettuce, corn, cucumber, carrot, beet, broccoli, and cauliflower. The selection was based on national consumption data from the Chilean Food Consumption Survey (ENCA) and was further validated through consultations with school food service staff from institutions representing different socioeconomic backgrounds. To ensure ecological validity, the vegetables were prepared using formats that reflected typical serving practices in Chilean households and school canteens (see Table 2). Preparation methods replicated those typically used in Chilean households and school canteens. The research team defined the procedures in collaboration with school food service staff to ensure that samples reflected children’s familiar eating contexts. Detailed information on preparation techniques and serving procedures is available in the methodological description of the first-stage study reported in Estay and Escalona (2025) [22].

### 2.3. Sensory Evaluation Procedure

Prior to formal data collection, the sensory evaluation protocol was piloted with a small group of children to ensure the feasibility and age-appropriateness of the procedures. Sensory evaluations were then conducted in small groups of 8 to 12 children over two consecutive sessions. Each child was seated individually, with sufficient spacing to minimize peer influence. Evaluations were conducted in all participating schools at approximately the same time of day—after the second midday recess—to standardize tasting conditions. Sessions lasted approximately 20 min and were scheduled between breakfast and lunch to minimize potential hunger- or satiety-related biases.

At the start of each session, children received clear, age-appropriate instructions from the main researcher and completed the evaluation independently using digital tablets (Qualtrics XM), with assistance from trained staff. Vegetable samples were served monadically in coded, individual containers and presented in a randomized order balanced using a Williams Latin square design to control for order and carryover effects.

Children rated each sample using a 7-point hedonic scale combining facial icons and numerical values, followed by two separate Check-All-That-Apply (CATA) tasks: one assessing sensory attributes (via words) and another capturing emotional responses (via emojis). In both cases, children were instructed to select all the terms or emojis that applied to their experience with the sample.

Overall liking was assessed using a validated 7-point facial hedonic scale. This format was chosen because it is appropriate for school-aged children and provides sufficient discriminatory power, and because the 7-point structure is familiar to Chilean children through the national school grading system, facilitating comprehension and consistent use of the scale.

For the CATA task, sensory and emotional attributes were presented in a fixed order to facilitate their use by children during the evaluation. This decision aimed to support reading and comprehension, particularly among children from vulnerable groups, some of whom required assistance from research assistants due to limited reading skills. Using a fixed list also helped participants more easily review and recognize all attributes when evaluating a second sample.

The sensory CATA list was developed through nine focus groups conducted with Chilean children from Low (*n* = 14), Medium (*n* = 15), and High (*n* = 14) socioeconomic backgrounds in Santiago. Each group included four to six fourth-grade children from the same schools participating in the main study, but from the previous academic year, yielding a total of 43 participants. Sessions were conducted at the schools during class hours and moderated by a trained facilitator following a structured discussion guide. Children were invited to describe a series of familiar vegetables displayed on a table, one sensory dimension at a time—appearance, aroma, taste, and texture. Laminated cards showing photographs of the vegetables were used to support comprehension and discussion.

Terms were selected based on frequency and clarity, and similar descriptors were merged through consensus among the research team, following procedures recommended for the development of consumer-derived CATA lists [28,33]. This participatory approach ensured that the final list captured the children’s natural perceptual language while remaining sensitive to subtle sensory differences across samples. The resulting sensory CATA included 13 descriptors—“sweet”, “sour”, “salty”, “bitter”, “hard”, “soft”, “juicy”, “crunchy/crispy”, “mild aroma”, “strong aroma”, “mild flavor”, “strong flavor”, and “fresh”—an appropriate number for use with children, as recommended in previous studies [28]. The list of descriptors was presented in a fixed order to facilitate reading and comprehension during the evaluation.

The emotional CATA was based on a set of 33 emoji descriptors corresponding to the validated list proposed by Schouteten et al. (2018) for assessing food-related emotions in children [29], which are summarized in Table 3. Following recent recommendations for using age-appropriate emotional tools with cross-cultural robustness [30], this approach provided a reliable and intuitive means of capturing affective responses in young participants.

All sensory tasks were designed to be completed within typical classroom attention spans and were administered using age-appropriate assessment tools and response formats to ensure comprehension and engagement.

### 2.4. Data Analysis

Data were analyzed using XLSTAT (version 2023.3, Lumivero, New York, NY, USA) and R Studio (version 2024.04.2, Boston, MA, USA). CATA data were coded as binary matrices (1 = selected; 0 = not selected). Cochran’s Q tests were applied independently to each sensory and emotional attribute to identify differences in citation frequency among vegetable samples. Pairwise comparisons were conducted using the Marascuilo procedure. Analyses were performed for the full dataset and separately for each socioeconomic group (Low, Medium-Low, Medium, Medium-High, and High); attributes with *p* < 0.05 were considered significantly discriminant.

To explore socioeconomic variation in perceptual and emotional discrimination, the number of significant attributes (*p* < 0.05) identified within each GSE group was used as a summary indicator of discriminative ability. These values were then correlated with socioeconomic level (treated as an ordinal variable from 1 to 5) using Spearman’s rank correlation, conducted separately for sensory and emotional attributes.

Correspondence Analyses (CA) were performed on aggregated attribute-by-product matrices to visualize associations between vegetables and descriptors, using chi-square distances as similarity measures. Finally, mean impact analyses were applied to evaluate the contribution of each sensory and emotional attribute to overall liking, identifying “must-have”, “must-not-have”, and “neutral” characteristics.

## 3. Results

### 3.1. Sensory Attributes Selection and Discriminative Patterns Across Socioeconomic Groups

A total of 13 sensory descriptors were included in the CATA questionnaire. When considering all participants together, every attribute was selected by more than 10% of children and showed significant discrimination among the eight vegetable samples according to Cochran’s Q tests (*p* < 0.05). Among the descriptors, crunchy/crispy was the least frequently selected overall, although it still exceeded 10% of selections in the full sample. Its frequency, however, fell below 10% among children from Low and Lower-middle socioeconomic groups, while remaining significant when considering the total sample (Table 4).

**Figure 1:**
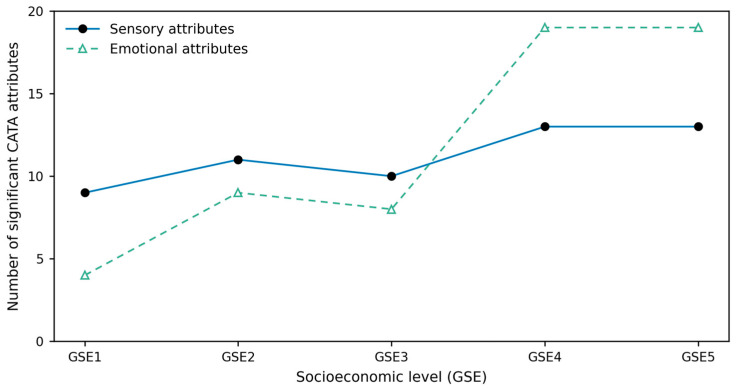
Number of significant sensory and emotional attributes selected by children across socioeconomic levels. Line plot showing the number of sensory (solid line with circles) and emotional (dashed line with triangles) descriptors significantly associated with vegetable samples (Cochran’s test, *p* < 0.05), analyzed by children (n = 363) across socioeconomic groups: low (GSE 1, *n* = 65), lower-middle (GSE 2, *n* = 82), middle (GSE 3, *n* = 66), upper-middle (GSE 4, *n* = 77), and high (GSE 5, *n* = 73).

When the data were analyzed by socioeconomic group (GSE), distinct response patterns emerged. Children from the lowest socioeconomic levels (Low to Lower-middle) selected fewer significant sensory descriptors (mean = 10) compared with those from higher groups (Upper-middle to High, mean = 13) (Figure 1). Low socioeconomic groups presented five attributes that did not reach significance in the Cochran’s Q test (bitter, crunchy/crispy, strong flavor, mild flavor, and mild aroma). For the Middle socioeconomic group, three sensory attributes were non-significant (mild aroma, mild flavor, and mild flavor). In contrast, all descriptors were significant for Upper-high and High socioeconomic groups.

### 3.2. Emotional Attribute Selection and Expression Patterns

The emotional CATA questionnaire included 33 emoji descriptors. When data from all socioeconomic groups were analyzed together, each emoji was selected by more than 10% of children to characterize the vegetable samples. However, six emojis: 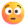, 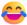, 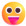, 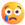, 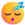, and 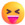 did not show significant differences across samples (*p* > 0.05).

When data were analyzed by socioeconomic groups, children from the lowest socioeconomic levels (Low to Lower-middle) selected fewer significant emotional descriptors (mean = 6.5) compared with those from higher groups (Upper-middle to High, mean = 19) (Table 5). In the lower socioeconomic groups, 13 emotional descriptors were non-significant (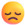, 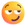, 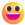, 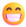, 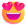, 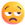, 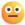, 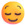, 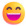, 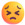, 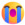, 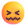, and 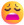). For the Middle socioeconomic group, 8 emojis were non-significant in discriminating among samples (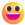, 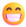, 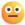, 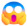, 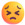, 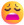, 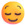, and 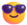). In contrast, for the higher socioeconomic groups (Upper-middle and High), all emojis used were significant. This pattern mirrored that observed for sensory descriptors, suggesting greater emotional differentiation and expressiveness among children from higher socioeconomic backgrounds.

The number of significant attributes per GSE increased progressively across socioeconomic levels (Figure 1), a trend that was evident for both sensory and emotional descriptors but more pronounced for the emotional ones. A very strong positive association was observed between socioeconomic level and the overall number of significant attributes (sensory and emotional combined; Spearman’s ρ = 0.97, *p* = 0.005). This result indicates that children from higher socioeconomic backgrounds more effectively differentiated and described vegetables both in sensory and emotional terms (Figure 1).

### 3.3. Sensory and Emotional Spaces and Their Relationship with Overall Liking

**Figure 2:**
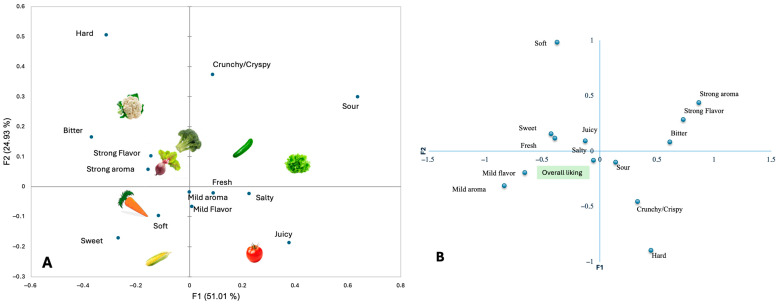
Sensory maps of familiar vegetables derived from Correspondence Analysis. (**A**) Associations between sensory CATA descriptors and vegetable samples based on all children’s selections (*n* = 363). (**B**) Projection of children’s overall liking as a supplementary variable onto the same sensory space. Data are aggregated across socioeconomic groups. The first two dimensions explain 77.75% of the total inertia (F1 = 55.56%, F2 = 22.19%).

**Figure 3:**
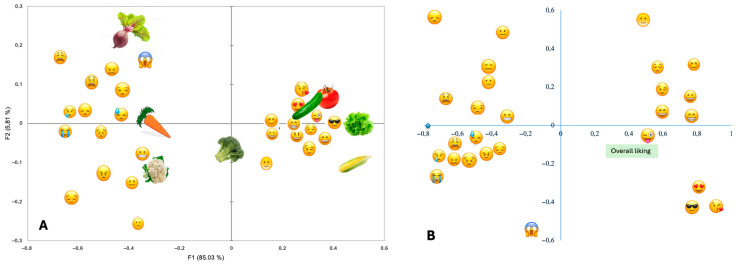
Emotional maps of familiar vegetables derived from Correspondence Analysis. (**A**) Associations between emoji-based emotional descriptors and vegetable samples based on all children’s selections (*n* = 363). (**B**) Projection of children’s overall liking as a supplementary variable onto the same emotional space. Data are aggregated across socioeconomic groups. The first two dimensions explain 94.74% of the total inertia (F1 = 85.03%, F2 = 9.67%).

Separate correspondence analyses (CA) were performed for the sensory and emotional CATA datasets to visualize how vegetable samples, descriptive terms, and overall liking were interrelated. For each domain (sensory and emotional), two plots are shown: one depicting the configuration of vegetables and emotional or sensory descriptors, and another displaying the projection of overall liking as a supplementary variable (Figure 2 and Figure 3).

The sensory correspondence analysis (CA) explained 75.94% of the total inertia (F1 = 51.01%, F2 = 24.93%) (Figure 2A). The sensory attributes that characterized the most liked samples were fresh, juicy, mild flavor, and mild aroma, whereas those that characterized the least liked samples were strong aroma, strong flavor, and bitter. When overall liking was projected onto the sensory space (Figure 2B), it was located near the descriptors fresh, juicy, salty, sweet, mild aroma, and mild flavor, indicating that these attributes were closely associated with higher appreciation. Conversely, bitter, strong aroma, and strong flavor were negatively associated with liking.

The emotional CA accounted for 90.84% of the total inertia (F1 = 85.03%, F2 = 5.81%) (Figure 3A). Positive emojis such as 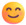, 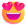, 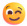, and 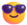 were grouped near vegetables associated with higher liking (tomato, corn, lettuce, and cucumber), whereas negative emojis (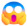, 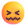, 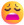, and 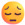) appeared close to less liked vegetables (beetroot). For cauliflower, neutral emojis (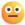 and 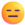) were selected. When overall liking was projected onto the emotional space (Figure 3B), it was located in the region dominated by positive emojis, suggesting that positive affective responses were systematically aligned with higher preference.

### 3.4. Contribution of Sensory Emotional Descriptors to Hedonic Responses

Mean Impact Analyses were performed to quantify how the presence of specific sensory and emotional descriptors influenced children’s overall liking scores. This method compares the mean hedonic ratings between cases in which each descriptor was selected and those in which it was not, with positive values indicating an increase in liking and negative values indicating a decrease.

When all data were analyzed together, the sensory attributes juicy (+0.6), mild flavor (+0.6), and fresh (+0.5) produced the greatest increases in hedonic ratings, followed by mild aroma (+0.4) and salty (+0.3). In contrast, strong aroma was associated with a decrease in liking (−0.5). Overall, these results indicate that mild (flavor and aroma), fresh, salty, and juicy characteristics exerted the strongest positive influence on children’s sensory preference profiles.

When the Mean Impact Analysis was conducted by socioeconomic group, clear differences emerged in how sensory descriptors influenced hedonic ratings. In the Low SES group, only the selection of juicy (+0.6) was associated with higher liking. In the Lower-middle SES group, juicy (+0.8), fresh (+0.7), and sweet (+0.6) were related to increased liking. A similar pattern was observed in the Middle SES group, where fresh (+0.8), juicy (+0.5), and sweet (+0.4) increased liking, while strong aroma (−0.6) decreased it. In the Upper-middle SES group, mild flavor (+0.7), mild aroma (+0.5), and juicy (+0.5) were linked to higher liking, whereas strong flavor (−0.5) and strong aroma (−0.7) were associated with a decrease. Finally, in the High SES group, juicy (+0.8), mild flavor (+0.7), fresh (+0.7), mild aroma (+0.6), and salty (+0.5) increased liking, while strong flavor (−0.5) and strong aroma (−0.6) were related to a decrease in liking.

For emotional descriptors, when all SES groups were analyzed together, four positive emojis: 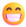 (+1), 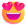 (+0.9), 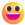 (+0.9), and 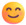 (+0.7) were associated with an increase in liking, indicating that their selection corresponded to higher mean hedonic scores. This pattern reflects a consistent co-occurrence between positive emotional expressions and increases in liking.

When the mean impact analysis was conducted by socioeconomic group, clear differences emerged. In the Low and Lower-middle SES groups, no emojis showed a significant impact on liking. The same was observed for the Middle SES group, where none of the emojis significantly affected hedonic scores. In contrast, in the Upper-middle SES group, four emojis: 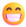 (+0.8), 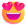 (+0.7), 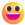 (+0.6), 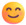 (+0.6), were associated with an increase in liking. In the High SES group, five emojis: 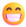 (+1.5), 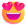 (+1.4), 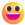 (+1.3), 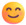 (+0.1), 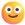 (+0.6) showed a positive impact, indicating that their selection corresponded to higher mean liking scores.

## 4. Discussion

This study provides novel evidence on how children describe and emotionally respond to familiar vegetables, and how these representations relate to their hedonic evaluations, highlighting the added value of combining sensory and emotional profiling beyond liking alone. It specifically highlights the role of socioeconomic background in shaping children’s ability to describe vegetables in sensory and emotional terms.

### 4.1. Socioeconomic Differences in Children’s Descriptive Ability

Socioeconomic context emerged as a key factor shaping children’s ability to describe and differentiate between vegetables. Children from higher socioeconomic groups used a greater number of sensory and emotional descriptors and showed a higher proportion of significant attributes in the CATA task, suggesting greater discriminative ability and lexical accessibility when characterizing familiar foods. These could be explained by reported data showing that linguistic and sensory richness in food descriptions increases with exposure, dietary diversity, and family food practices [28,34,35,36].

Children from lower socioeconomic backgrounds, in contrast, tended to rely on a narrower set of terms. This pattern may reflect differences in everyday exposure to vegetables, as well as limited opportunities to engage in sensory and verbal interactions around food at home and school, as shown in studies on sensory education and family food practices [35,36,37].

Notably, however, these socioeconomic contrasts in descriptive and emotional discrimination did not translate into differences in liking scores for the same vegetable samples [22]. While hedonic evaluations remained comparable across socioeconomic groups, the CATA data revealed marked divergences in how children perceived and emotionally represented the vegetables. This finding underscores the capacity of sensory- and emoji-based CATA approaches to capture more nuanced and multidimensional aspects of children’s food experiences that remain invisible through hedonic measures alone. Similar conclusions were drawn by Tan et al. (2024), who found that even when children’s liking scores remained relatively stable, their emotional responses and willingness to eat showed meaningful variation, underscoring that emotion-based measures capture nuances in children’s food experiences that are not reflected in hedonic ratings alone [38].

Taken together, these results support the idea that sensory and affective literacy, children’s capacity to verbalize and interpret their own food experiences, is unevenly distributed across socioeconomic contexts. This disparity may not only influence how children describe vegetables, but also how they perceive and experience vegetable consumption. Recognizing these differences may help to better tailor vegetable promotion strategies, particularly in more vulnerable socioeconomic contexts.

### 4.2. Sensory and Emotional Dimensions of Children’s Responses Across SES

Across socioeconomic groups, children’s sensory and emotional descriptions revealed distinct but complementary patterns. Sensory attributes captured how children perceived and differentiated the vegetables, while emojis reflected their affective engagement. Both dimensions are interconnected aspects of children’s experience with food.

The attributes *bitter*, *strong flavor*, *mild flavor*, and *mild aroma* were among those that failed to discriminate between samples in the lower socioeconomic groups. Among these, *bitterness* did not reach significance only in the Low SES group. The difficulty in using *bitterness* as a discriminative attribute was not unexpected, as previous research has shown that even adult consumers often struggle to detect or correctly identify bitter compounds—particularly at low or moderate intensities [39]. Moreover, the intensity of the *bitter* attribute in the vegetables evaluated was not particularly salient, which may have further reduced its perceptual distinctiveness. Children’s ability to use this descriptor is influenced by the perceptual salience of bitterness, their previous exposure to bitter-tasting vegetables, and the developmental processes through which flavor learning occurs [18,40].

Intensity-related descriptors—such as *strong* or *mild* flavor and aroma—were consistently challenging for children from lower and middle socioeconomic backgrounds. This may reflect the fact that olfactory cues, which largely underlie these perceptions, are inherently more difficult to access consciously and to describe compared with gustatory or textural cues [41]. Indeed, the limited use of odor vocabulary in everyday life—even among adults—restricts the development of olfactory awareness and descriptive ability, ultimately making smells less consciously perceived and more difficult to articulate [41]. By contrast, in the upper-middle and high SES groups, all sensory attributes were significant, indicating a more discriminative use of sensory vocabulary and possibly a higher familiarity with the evaluated vegetables. Overall, these findings reinforce the notion that sensory modalities develop at different rates and are differentially shaped by exposure and cultural experience.

Among sensory descriptors used, *crunchy*/*crispy* was the least frequently used across all socioeconomic groups, which is consistent with the sensory characteristics of the vegetable set—among the eight samples evaluated, only lettuce presented a clearly crisp texture. In addition, although the sensory vocabulary was developed with children from the same population through focus groups, *crunchy*/*crispy* may have been less intuitive to apply during tasting sessions compared with the other texture-related terms (*hard* and *soft*), which were simpler and conceptually opposite.

Beyond the number of descriptors, clear patterns emerged in the emotional responses associated with vegetable liking. Across socioeconomic groups, children use positive emojis when describing vegetables they liked and negative or neutral ones for those they liked less. Positive emotions—such as 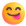, 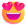, or 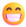
—were strongly associated with higher hedonic ratings, reflecting pleasant affective engagement, whereas negative or low-arousal emotions were linked to lower liking. This correspondence between emotional valence and hedonic evaluation supports the idea that emoji-based measures can effectively capture children’s affective reactions to food in an intuitive and age-appropriate way [29,42].

Some emojis did not discriminate among samples, even when all socioeconomic groups were considered together. These included 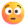, 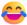, 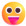, 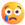, 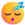, and 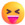. Their low discriminative power may indicate that these expressions represent more ambiguous or context-independent reactions rather than specific affective responses to the vegetables themselves. For example, 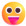 and 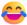 may capture playful or socially driven reactions, while 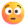 and 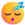 denote confusion or low arousal states that are less directly linked to taste evaluation. Similar patterns have been reported in previous studies using emoji-based emotion measures with children, where low-arousal or mixed-valence emotions show weaker associations with specific food stimuli [29,42,43]. These findings suggest that children’s emotional responses to food are organized primarily around clear positive and negative valence cues.

### 4.3. Emotional and Sensory Correlates of Preference

The mean-impact analyses revealed that children’s liking of vegetables was systematically aligned with both sensory and emotional responses. Overall, the attributes juicy, fresh, and mild flavor produced the strongest increases in hedonic ratings, while strong aroma was associated with a decrease in liking. These findings indicate that pleasant sensory experiences in children are characterized by moderate intensity, a feature consistent with their developmental preference for mild and less complex stimuli [36,44,45].

The recurrent association of juicy and fresh with higher liking scores indicates that these attributes play a central role in children’s sensory appreciation of vegetables. Their consistent positive impact suggests that children perceive these qualities as markers of palatability and pleasant sensory experience. Similarly, the positive association of mild flavor with liking reflects a preference for balanced, non-pungent tastes—foods that avoid bitterness or strong aromatic notes tend to feel more familiar and safe, facilitating acceptance [46]. Conversely, the negative contribution of strong aroma reinforces the notion that olfactory intensity can act as a barrier to acceptance, particularly for vegetables containing sulfurous or earthy volatiles, which are known to elicit rejection even in familiar contexts [46].

On the emotional dimension, positive emojis such as 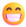, 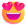, 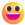, and 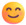 showed the strongest association with increased liking, indicating a clear correspondence between hedonic pleasure and positive affective expression. Interestingly, no negative or ambiguous emojis showed a significant effect on liking—either in the combined analysis or within socioeconomic groups—suggesting that expressions of rejection, confusion, or playfulness (e.g., 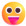, 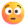, 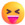) may represent peripheral or socially driven reactions rather than direct hedonic evaluations. This pattern reinforces that only positive affective cues contribute meaningfully to hedonic appraisal in this age group, consistent with previous findings that children’s emotional lexicon for food is dominated by expressions of liking and enjoyment [29,42].

The convergence between sensory and emotional predictors of liking underscores the intertwined nature of perception and affect in children’s food experience. Attributes such as juicy, fresh, and mild flavor evoked positive emotions such as 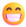 and 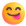, indicating that sensory comfort and positive affect jointly shape vegetable acceptance. As noted by Tan et al. (2024), emotional responses can vary even when liking remains relatively stable, revealing perceptual nuances that hedonic scores alone do not capture—a pattern also evident in the present study, where the alignment between sensory and emotional dimensions provided a richer understanding of children’s preferences beyond liking alone [38]. The combined use of sensory and emoji-based CATA methods thus offers a powerful framework for capturing both perceptual and affective dimensions of food experience in children, bridging the gap between what they taste and how they feel about it.

### 4.4. Study Limitations and Strengths

Although the emotional descriptors (emojis) used in this study were not directly generated by the participating children, they were derived from validated sets previously applied in child-centered food studies [29,42,43]. The use of emoji-based measures represents a methodological advantage, as visual symbols minimize linguistic and cognitive barriers, allowing children to express emotions intuitively without the need for verbal explanation [29,42]. While the use of an extensive emoji list enabled a nuanced exploration of children’s emotional responses, future research may assess the performance of reduced or task-specific emoji subsets depending on study objectives.

In contrast, the sensory descriptors were developed through focus groups conducted with children from the same population, which constitutes a major strength of the study. This participatory approach ensured that the sensory vocabulary reflected children’s own language and conceptual understanding, improving ecological validity and engagement during tasting sessions. The combination of validated emoji tools with child-generated sensory descriptors thus represents a balanced methodological design that maximizes both reliability and child-centered relevance.

Moreover, the inclusion of children from all socioeconomic levels within a single national context constitutes another key strength of this work. Chile presents one of the highest levels of socioeconomic inequality among OECD countries [47], making it an especially relevant setting for examining how social and economic disparities shape sensory–affective responses to food. The marked contrasts in dietary exposure, food environments, and cultural practices across Chilean socioeconomic groups provided a unique opportunity to observe how sensory and emotional literacy develop in children under differing life conditions.

## 5. Conclusions

This study provides novel insights into how children perceive and emotionally respond to familiar vegetables, revealing that these experiences are deeply influenced by socioeconomic background. Through the combined use of sensory and emoji-based CATA approaches, the findings demonstrate that, while hedonic evaluations of vegetables remained comparable across socioeconomic groups, clear differences emerged in children’s descriptive and emotional repertoires.

Children from higher socioeconomic backgrounds exhibited greater sensory and affective literacy—expressed through a richer and more discriminative use of sensory and emotional descriptors— suggesting that this may reflect broader differences in exposure, dietary diversity, and opportunities for verbal interaction around food. In contrast, children from lower socioeconomic groups relied on a narrower and less discriminative set of descriptors, reflecting structural disparities in opportunities for sensory exploration and food-related dialogue. These results emphasize that sensory and emotional competence in food contexts is not innate but socially constructed through experience and interaction.

Importantly, the divergence in descriptive and emotional abilities was not mirrored in liking scores, indicating that hedonic appreciation alone does not capture the full spectrum of children’s engagement with familiar vegetables. This highlights the value of integrating sensory and affective dimensions to achieve a more comprehensive understanding of food acceptance in childhood. Practically, the results suggest that interventions aiming to promote vegetable acceptance—particularly in lower-SES contexts—should focus not only on exposure but also on fostering children’s ability to perceive, describe, and emotionally connect with foods.

Overall, these findings advance the notion that children’s relationship with vegetables is shaped as much by sensory and emotional literacy as by taste itself, pointing to the importance of addressing both perceptual and social dimensions in strategies to promote healthier and more equitable eating behaviors.

Future research should explore how emotional representations of vegetables evolve across developmental stages and examine whether targeted sensory or educational interventions can enhance children’s expressive and affective engagement with vegetables, particularly in lower socioeconomic contexts.

## Figures and Tables

**Table 1:** Sociodemographic profile of participating children (*n* = 363).

Variable	Category	*n*	%
SES *	Low	65	17.9
	Low-medium	82	22.6
	Medium	66	18.2
	Medium-high	77	21.2
	High	73	20.1
Sex	F	193	53.2
	M	170	46.8

* Socioeconomic status and sex: child’s sex.

**Table 2:** Description of vegetable samples and serving conditions.

Vegetable	Preparation/Serving Condition	Serving Temperature
Lettuce	Raw and slightly seasoned with salt, lemon, and sunflower oil	Room temperature
Tomatoes	Raw and slightly seasoned with salt and sunflower oil	Room temperature
Corn	Boiled with salt and slightly seasoned with sunflower oil	Room temperature
Cucumber	Raw and slightly seasoned with salt, lemon, and sunflower oil	Room temperature
Broccoli	Boiled with salt	Warm
Carrots	Boiled with salt	Warm
Cauliflower	Boiled with salt	Warm
Beets	Boiled and slightly seasoned with salt, lemon, and sunflower oil	Room temperature

**Table 3:** Emoji-based emotional descriptors and valence classification used in the emotional CATA task *.

Emoji	Valence Classification
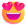 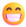 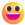 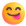 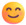 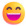 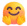 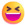 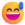 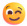 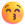 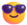 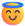	Positive
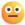 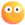	Neutral
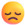 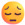 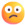 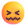 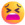 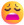 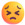 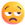 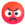 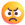 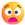 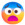 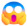 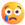 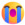 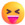	Negative
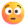	Negative/mixed
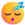	Negative/low arousal

* Based on the validated emoji-based emotion list proposed by Schouteten et al. (2018) [29]

**Table 4:** Selection frequency and significance of sensory descriptors across socioeconomic groups (GSE).

GSE	Descriptors Selected by <10% of Children (Within GSE)	Descriptors Not Significantly Discriminant (Cochran’s Q, *p* < 0.05)
All	None *	None
Low	Crunchy/Crispy	Bitter, Crunchy/Crispy, Strong flavor, Mild flavor
Lower-middle	Crunchy/Crispy	Mild aroma, Mild flavor
Middle	None	Mild aroma, Mild flavor, Strong flavor
Upper-middle	None	None
High	None	None

* “None” indicates that, within that socioeconomic group, all descriptors exceeded the 10% citation threshold and were statistically significant according to Cochran’s Q test (*p* < 0.05).

**Table 5:** Selection frequency and significance of emotional descriptors using emojis across socioeconomic groups (GSE).

GSE	Descriptors Selected < 10%	Descriptors Not Significant
**All**	None	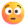 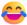 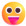 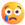 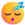 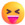
**Low**	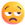 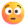 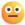 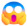 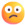	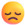 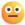 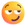
**Lower-middle**	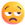 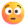 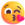 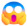 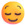 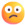	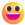 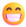 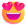 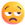 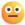 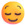 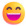 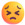 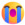 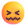 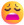
**Middle**	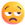 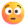 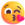 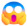 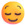 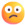	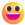 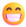 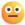 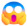 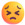 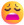 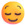 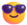
**Upper-middle**	None	None
**High**	None	None

## Data Availability

The data presented in this study are available on request from the corresponding author. The data are not publicly available due to privacy restrictions.
